# Addiction Problems, Aggression, and Quality of Life in People with Different Occupations in South Korea

**DOI:** 10.3390/healthcare9020141

**Published:** 2021-02-01

**Authors:** Hwee Wee, Gweon-Young Kang

**Affiliations:** 1 Department of Nursing, Kunsan National University, Gunsan-si 54150, Korea; 2 Department of Psychiatry Clinic, Gunsan Medical Center, Gunsan-si 54105, Korea; halfmoony@hanmail.net

**Keywords:** alcohol misuse, addiction, gambling, aggression, quality of life

## Abstract

Addiction is related to aggression and quality of life. This study examined the relationship between these three factors according to occupation group in a mixed urban/rural area to better understand adult addiction problems. This study was a secondary data analysis of cross-sectional data collected by a 2017 regional survey of adults living in Gunsan City, South Korea. The survey included 500 people split into the unemployed (Group1), full-time homemakers (Group2), and primary (Group3), secondary (Group4), and tertiary (Group5) industry workers. Addiction problems and aggression were positively correlated (*p* < 0.01). Aggression and alcohol use disorder were correlated in Group3 (r = 0.31), Group4 (r = 0.34), and Group5 (r = 0.32), and aggression and smartphone addiction were correlated in Group2 (r = 0.39) and Group4 (r = 0.31). Problem gambling was correlated with aggression in Group5 (r = 0.39). A negative relationship between quality of life and alcohol use disorder occurred in Group1 (r = −0.36). According to the occupation group, the relationships between addiction problems, aggression, and quality of life were different. These findings suggest that addiction management for adults should be implemented in consideration of occupation groups.

## 1. Introduction

Substance misuse and behavioral addiction are serious health problems. Approximately three million people die each year because of harmful use of alcohol [1]. The burden of diseases and injuries caused by alcohol consumption is so severe that society cannot ignore it. In addition, some people involved in gaming and gambling behavior using devices such as smartphones may develop disabilities related to functional impairment or distress due to their addictive behavior [1].

Addiction is not only a health problem but also a factor associated with aggression [2] and violence [3]. Alcohol use may cause violence-related aggression [4,5], and problem gambling can lead to aggression [6]. Moreover, problematic smartphone use is also related to aggression and hostility [7]. Aggression includes various behaviors but is mostly associated with violent behaviors such as fights and quarrels. It is defined as behavior aimed at harming others via words, body parts, or tools [8]. Aggression damages others and requires serious attention from society, so it must be considered when a community manages addiction problems. In South Korea, the national mental health project’s policy goals include “minimizing health impairment and social damage caused by addiction” [9].

Moreover, people with addiction problems have lower quality of life (QOL) [10]. QOL is significantly impaired in subjects with alcohol abuse problems [11], and smartphone addiction also negatively affects QOL [12]. Furthermore, gambling was negatively correlated with QOL [13]. The treatment of addiction aims at recovery, which is defined as abstinence and QOL improvement. In the treatment of and recovery from addiction problems, it is very important to integrate QOL into research and available services [14]. QOL is a multidimensional complex concept for measuring people’s living standards and status beyond individual perception. QOL is an important indicator of urban and rural economic development and the level of social civilization and is directly related to well-being [15].

There is a relationship between occupation engagement, health, and well-being [16]. Occupations contribute to what individuals perceive as meaningful, their relationships with others, and the structure of their daily lives. However, addiction may meet these needs when social, political, or other factors prevent an individual from engaging in a satisfactory occupation [17].

In a few studies, differences in alcohol misuse, smartphone addiction, and problem gambling were reported according to occupation groups [18,19,20,21]. Farm laborers were more likely to be current drinkers than individuals in other occupations and binge than others [19]. Smartphone addiction had a job difference between a university student and a professional [20]. It was reported that problem gambling appears differently by occupation group [21]. However, the classification of occupation groups was not the same. Clark [22] divided the industrial structure into primary (defined as agriculture, forestry, and fishing), secondary (defined as manufacturing, mining, and building), and tertiary (include commerce, transport, services, and other economic activities) industries. Additionally, the occupation situation of adults should take into account unemployed and full-time homemakers.

It is necessary to determine whether there are differences in addiction problems and related variables depending on an individual’s occupation and determine how the relationships vary according to occupation group. Therefore, this study was concerned with understanding addiction problems, aggression, and QOL of residents in mixed urban/rural areas by occupation. The objectives of the research were to (1) determine the differences between alcohol use disorder, smartphone addiction, problem gambling, aggression, and QOL according to occupation groups, (2) identify the relationships between alcohol use disorder, smartphone addiction, gambling problem, aggression, and QOL by occupation group.

## 2. Materials and Methods

### 2.1. Sample

This study was based on data from surveys conducted by the Addiction Management Center (AMC) in Gunsan City. We received approval to use this survey data in our analysis. Gunsan City is a mixed urban/rural area with a population of 267,987, located in Jeollabuk-do, South Korea [23]. Data were collected between May and August 2017. The participants of the survey were adults aged over 19 residing in Gunsan City, categorized into five groups of 100 people each. These five groups comprised unemployed people (including university students who do not engage in economic activities, Group1), full-time homemakers (Group2), and workers in the primary (Group3), secondary (Group4), and tertiary industries (Group5). In this study, all 500 data points were selected as the final study participants. Survey participants had taken part in programs implemented by AMC and related organizations or had been contacted by investigators within the community area. They agreed to participate after receiving guidance on the study by AMC staff or investigators. Gunsan AMC only rarely included drug addiction in their optional programs, so only alcohol, smartphone, and gambling variables were investigated.

### 2.2. Measures

#### 2.2.1. Alcohol Use Disorder

This study used the Korean version of the World Health Organization (WHO) Alcohol Use Disorders Identification Test (AUDIT) [24] in Korean (K-AUDIT) [25]. This test contains a total of 10 questions scored on a 5-point Likert scale from 0 to 4 points (total scores range from 0 to 40). The cutoff is 8 points, and four risk levels can be classified according to score (Table 2). Higher scores indicate greater likelihood of hazardous and harmful drinking. In this study, Cronbach’s α was 0.88.

#### 2.2.2. Smartphone Addiction

The Adult Smartphone Addiction Proneness (ASAP) scale [26], developed to self-diagnose smartphone addiction among adults, was used. It has 15 questions (three of which are negatively framed) and consists of four subdomains: (1) disturbance of adaptive functions, (2) virtual life orientation, (3) withdrawal, and (4) tolerance. It is scored on a 4-point Likert scale ranging from 1 to 4 points (total scores range from 15 to 60). Users can be divided into three groups according to total score (Table 2). At the time of development, the reliability of the tool measured using Cronbach’s α was 0.81. In the current study, it was 0.85.

#### 2.2.3. Problem Gambling

The Korean version of the Problem Gambling Severity Index (KPGSI) [27] was used. This is a translation of the Problem Gambling Severity Index (PGSI) [28] and includes nine items for identifying the prevalence rate of problem gambling from the Canadian Problem Gambling Index (CPGI). It is scored on a 4-point Likert scale ranging from 0 points for “never” to 3 points for “almost always” (total scores range from 0 to 27). The problem risk can be classified into four groups according to total score (Table 2). In this study, Cronbach’s α was 0.91.

#### 2.2.4. Aggression

A Korean adaption of Buss and Perry’s Aggression Questionnaire (AQ) [29,30] was used; it includes physical and verbal aggression, anger, and hostility. It consists of 27 questions and is scored on a 5-point Likert scale from 1 “not at all” to 5 “very much” (total scores range from 27 to 135). In this study, Cronbach’s α was 0.86.

#### 2.2.5. Quality of Life

The Korean version of the WHO Quality of Life Assessment Scale [31] was used. WHO developed the QOL Assessment Instrument (WHOQOL-BREF) [32] for use in epidemiological studies. It includes the concepts of physical health and psychological health, social relationships, and environment. It has a total of 26 questions, of which three are negatively framed. It is scored on a 5-point Likert scale. QOL score was calculated according to the WHOQOL-BREF assessment form [32]. The domain scores were converted into a transformed score of 4–20 points. In this study, Cronbach’s α was 0.93.

### 2.3. Data Analysis

Data were analyzed using SPSS version 24.0. Descriptive statistics were used to assess participants’ general characteristics. Differences in variables were analyzed according to occupation using one-way analysis of variance (ANOVA). Age is a variable related to addiction and behavior. Therefore, we repeated the analysis of covariance (ANCOVA), which treats age as a covariate. ANCOVA was performed after confirming normality, homogeneity of variances, and homogeneity of regression slopes, and we included significance level and effect size (partial η^2^) in the analysis. We used the Bonferroni method as a post hoc test after ANCOVA, and the relationship between the variables was analyzed using the Pearson correlation coefficient. In order to detect a Pearson’s correlation coefficient of *r* = 0.30 with 80% power (alpha = 0.05, two-tailed), G*Power 3.1.9.4. suggested we would need 84 participants [33]. One hundred samples were included in each of the five occupational groups.

### 2.4. Ethical Considerations

This study was a secondary data analysis of cross-sectional data collected by a 2017 regional survey of adults living in Gunsan City, South Korea. The study was reviewed and approved for exemption by the Kunsan National University Institutional Review Board (IRB approval number: 1040117-202009-HR-019-01).

## 3. Results

### 3.1. Participant Characteristics

The general characteristics of the participants are shown in Table 1. Of these, 50.8% (*n* = 254) were female and 49.2% (*n* = 246) were male. Their average age was 47.3 ± 16.53 years, and 41–50 years old was the most common age range (*n* = 129, 25.8%). A total of 68.0% (*n* = 340) were residents in urban areas and 25.8% (*n* = 129) were residents in rural areas.

### 3.2. Differences between Occupational Groups

Table 2 presents the percentage by risk level to identify problematic alcohol use disorder, smartphone addiction, and problem gambling in each group. For alcohol use disorder, in Group4, 48% (close to half of the total) had a cutoff score of 8 or more. In Group1, 10% of participants had a smartphone addiction score of 40 or higher, and 12% had at least a low level of problem gambling.

Table 3 shows the observed means of each group’s variables and the age-controlled adjusted means. In addition, there were no significant differences in problem gambling, aggression, or QOL of physical health in the ANCOVA results.

The differences between groups of each variable were significant for alcohol use disorder (F = 3.63, *p* = 0.006, partial η^2^ = 0.03), smartphone addiction (F = 4.08, *p* = 0.003, partial η^2^ = 0.04), overall QOL (F = 2.71, *p* = 0.029, partial η^2^ = 0.02), psychological health (F = 5.51, *p* < 0.001, partial η^2^ = 0.04), social relationships (F = 3.39, *p* = 0.009, partial η^2^ = 0.03), and environment (F = 2.59, *p* = 0.036, partial η^2^ = 0.02) among the QOL domains.

Regarding AUDIT scores, the adjusted mean was the highest at 8.07 in Group4 and significantly higher than in Group2 on the Bonferroni test. ASAP’s adjusted mean was the highest at 28.47 in Group1 and significantly higher than in Group5. Group1 also had the highest PGSI and AQ scores, but this was not statistically significant. In the WHOQOL-BREF score, the adjusted mean of Group1 was the lowest at 3.24. Among the QOL domains, psychological health’s adjusted mean was the lowest at 12.24 in Group1 and significantly lower than in Group3, Group4, or Group5. Additionally, social relationship’s adjusted mean was the lowest at 12.94 in Group1 and significantly lower than in Group2 or Group3 on the Bonferroni test.

### 3.3. Correlation Analysis

Table 4 shows the relationships among alcohol use disorder, smartphone addiction, problem gambling, aggression, and QOL by occupational group. The *p*-value was less than 0.01, and the results with a median effect size (r > 0.30) [34] or higher in the relationships between variables were as follows:

In Group1, there was a negative correlation between QOL and alcohol use disorder (r = −0.36, *p* < 0.001). In Group2, smartphone addiction was positively correlated with aggression (r = 0.39, *p* < 0.001) and problem gambling (r = 0.31, *p* = 0.002). QOL was negatively correlated with aggression (r = −0.38, *p* < 0.001).

Alcohol use disorder was positively correlated with aggression (r = 0.31, *p* = *0*.002) and problem gambling (r = 0.43, *p* < 0.001) in Group3. In Group4, aggression was positively correlated with alcohol use disorder (r = 0.34, *p* = 0.001) and smartphone addiction (r = 0.31, *p* = 0.002). QOL was negatively correlated with aggression (r = −0.32, *p* = 0.002). In Group5, aggression was positively correlated with alcohol use disorder (r = 0.32, *p* = 0.001) and problem gambling (r = 0.39, *p* < 0.001).

## 4. Discussion

The urban–rural complex city is characterized by the coexistence of primary, secondary, and tertiary industries. This study was conducted to help manage addiction problems in such urban–rural complex communities, decrease aggression, and improve residents’ QOL. Peculiarities were found for each occupation group. Based on the results, community-based workplace addiction management to deal with residents’ aggression and QOL can be considered.

Aggression was highest in Group1 (adjusted M = 52.04) and lowest in Group3 (adjusted M = 49.27), but there was no statistically significant difference. Addiction problems and aggression were positively correlated. Alcohol use disorder and aggression were significantly correlated only in Group3, Group4, and Group5, and smartphone addiction was significantly correlated in Group2 and Group4. Problem gambling was correlated with aggression only in Group5. In Group1, which had the highest level of aggression, there was no correlation between aggression and addiction problems.

There was a significant difference in QOL between groups, and overall QOL was lowest in Group1 (adjusted M = 3.24) and highest in Group3 (adjusted M = 3.48). In the case of Group1, the score was the lowest for all four subdomains of QOL, and there were significant differences in psychological health, social relationships, and environment. Results of the post-test showed that all three domains were significantly lower than in Group3, and psychological health was significantly lower than in Group3, Group4, and Group5. Regarding the correlation between QOL and addiction problems, only Group1 showed a significant negative relationship between QOL and alcohol use disorder.

Group1 comprised unemployed people and college students who do not engage in economic activities. They had lower QOL and a higher level of smartphone addiction than other groups. However, QOL was only significantly correlated with alcohol use disorder. Increasing poverty increases alcohol use and alcohol problems, and recent unemployment reduces alcohol use, while long-term unemployment increases alcohol use [35]. Therefore, for those who have financial difficulties due to no job, rather than providing a simple addiction management program, practical and diverse community support is needed that considers psychological health, social relationships, and living environments. In addition, psychological support programs are needed to improve the perception of participants’ position in life and the definition of QOL [15]. One thing to consider is that workplace-based programs are not possible for such individuals because they do not have a job and spend their days in a variety of places. To improve their low level of social relationships, face-to-face or group programs are considered useful, and programs using community organizations or social support groups are suggested.

Group2 included full-time homemakers, mostly women and three men. This group’s results showed smartphone addiction scores second to those of Group1; aggression was positively correlated with smartphone addiction, and QOL was not correlated with addiction problems. These results were similar to those of Group3, Group4, and Group5, but not the unemployed group. Socially, “full-time homemakers” tend to be regarded as “people without a job or housewives”, but when it comes to addiction management, they should be regarded as “people who perform housework as their occupation”. However, they often spend their days at home, and the QOL of social relationships was relatively good, so programs using community organizations or individual support groups will be more effective for this group than workplace-based programs. Because smartphone functionality can vary with regard to information, entertainment, and communication [36], additional research is needed to confirm the characteristics of full-time homemakers’ use of smartphones and management programs should be developed accordingly. In addition, alternative treatments such as cognitive-behavioral approaches, motivational interviewing, exercise rehabilitation, therapeutic recreation, drumming therapy, and art therapy [37] can be used to manage smartphone usage problems in most groups. Since the use of smartphones to access information and communicate with others is expected to increase in the future, it is necessary to identify people’s smartphone usage patterns and help them use smartphones properly to minimize side effects.

Results for Group3 (workers in primary industries), Group4 (workers in secondary industries), and Group5 (workers in tertiary industries) showed that aggression was positively correlated with alcohol use disorder and QOL was not correlated with addictions. Therefore, an intervention program that reflects self-control for aggression and alcohol misuse should be implemented for workers in all occupation groups. Roman [38] suggested the implementation of a workplace employee assistance program for employee alcohol problem prevention. Workplace programs include primary and secondary prevention, but primary prevention is often more cost-effective than secondary prevention. However, it has been argued that it is difficult to deal with adult employees’ alcohol drinking problem in individual workplaces and that the employer should not be held responsible. Therefore, if a community addiction-specialized organization such as AMCs in South Korea provided services connected with the workplace, it will be possible to garner the benefits of a workplace-based program without the drawbacks of employer responsibility.

The relationship between aggression and problem gambling was significant in Group5. Excessive gambling can induce a broad range of physical, social, and economic problems at the individual and family level [39]. Unlike alcohol misuse and smartphone addiction, gambling involves betting on something of value (e.g., money), and it is common to lose money or even take debt from gambling activities. Poor economic times and economic distress can influence aggression at the individual level [40]. Group 5, including commerce and services dealing with direct capital, was sensitive to economic distress due to their occupational characteristics. Therefore, it is presumed that the correlation between problem gambling and aggression appeared significant. Further research is needed on the variables involved in the relationships between gambling and aggression in this group. Lakey [41] stated that gambling in excess causes various intrapersonal and interpersonal problems, that problems caused by severe gambling negatively correlate with dispositional mindfulness, and that mindfulness alleviates gambling problems and helps make appropriate decisions. Recently, the effects of limit-setting as a self-control strategy for people who are regularly gambling were reported, and it was proposed as a public health intervention [42]. Gambling has been included in medical diagnoses as a disorder since 2013, reflecting evidence that gambling behavior and drug abuse both activate the reward system and that behavioral symptoms due to disability are similar [43]. In addition to AMCs, South Korea established and has operated the Korea Center on Gambling Problems nationwide, focused on preventing and managing gambling problems only, since 2013 [44]. There is a need for better gambling problem management through cooperation between institutions.

Summarizing the results of this study, Group1 showed a correlation between QOL and alcohol misuse. Group2, Group3, Group4, and Group5 showed significant results in the relationships between alcohol misuse and aggression, Group2 and Group4 in the relationships between smartphone addiction and aggression, and Group5 in the relationship between problem gambling and aggression. Therefore, unlike other occupation groups, Group1 related QOL, rather than aggression, to an addiction problem, and addiction problems and aggression were related in all other groups. Based on these results, future research directions are suggested as follows: Since this study analyzed data from only one mixed urban/rural area, further studies will be needed to collect data from various communities and compare the results. In addition, since there may be differences in drinking patterns between urban and rural workers in mixed urban/rural areas [18], addiction management programs should be based on each residential areas’ characteristics. Further research is needed to obtain more evidence in this regard.

However, since only one part of AMC survey data in one mixed urban/rural area was analyzed, there are limitations as follows: (1) Non-evaluation and exclusion of participants’ mental disorders may have biased the results in aggression and QOL. (2) College students were included in the unemployed group; although students do not work, they may be involved in activities that require more time.

## 5. Conclusions

Substance misuse and behavioral addiction are serious problems related to mental health and QOL. This study analyzed secondary data collected by a regional survey of adults living in a mixed urban/rural area in South Korea to confirm the relationship between addiction problems, aggression, and QOL by occupation status. There were differences in the degree of alcohol use disorder, smartphone addiction, and QOL between unemployed, full-time homemaker, and primary, secondary, and tertiary industrial worker groups. The relationships between the variables were different for each group. The results could be used as basic data for developing adult workplace-based programs among addiction management projects to be implemented in the community. Therefore, it is suggested that addiction management programs should be planned and implemented in consideration of occupation groups.

In addition, since only one part of AMC survey data in one area was analyzed, there are limitations to the generalizability of these results; various communities should be researched in the future and the results should be compared to those found in this study.

## Figures and Tables

**Table 1:** Participants’ demographic data.

Variable	Category	Frequency (%)					
		Group1	Group2	Group3	Group4	Group5	Total
Gender	Female	55 (55.0)	97 (97.0)	29 (29.0)	14 (14.0)	59 (59.0)	254 (50.8)
	Male	45 (45.0)	3 (3.0)	71 (71.0)	86 (86.0)	41 (41.0)	246 (49.2)
Age (years)	≤30	45 (45.0)	4 (4.0)	0 (0.0)	20 (20.0)	11 (11.0)	80 (16.0)
	31–40	10 (10.0)	24 (24.0)	8 (8.0)	29 (29.0)	25 (25.0)	96 (19.2)
	41–50	13 (13.0)	34 (34.0)	11 (11.0)	32 (32.0)	39 (39.0)	129 (25.8)
	51–60	12 (12.0)	22 (22.0)	22 (22.0)	14 (14.0)	17 (17.0)	87 (17.4)
	61–70	12 (12.0)	14 (14.0)	28 (28.0)	4 (4.0)	6 (6.0)	64 (12.8)
	≥71	8 (8.0)	2 (2.0)	31 (31.0)	1 (1.0)	2 (2.0)	44 (8.8)
Residential area	Urban	70 (70.0)	86 (86.0)	21 (21.0)	73 (73.0)	90 (90.0)	340 (68.0)
	Rural	20 (20.0)	9 (9.0)	77 (77.0)	17 (17.0)	6 (6.0)	129 (25.8)
	Missing	10 (10.0)	5 (5.0)	2 (2.0)	10 (10.0)	4 (4.0)	31 (6.2)

Notes: The five groups comprised unemployed people (including university students who do not engage in economic activities, Group1), full-time homemakers (Group2), and workers in the primary (Group3), secondary (Group4), and tertiary industries (Group5).

**Table 2:** Level of addiction problem by group.

Variable	Score	Risk Level	Frequency (%)					
			Group1	Group2	Group3	Group4	Group5	Total
Alcohol use disorder	0–7	Low risk	69 (69.0)	82 (82.0)	72 (72.0)	52 (52.0)	70 (70.0)	345 (69.0)
	8–15	Risky level	18 (18.0)	11 (11.0)	20 (20.0)	29 (29.0)	19 (19.0)	97 (19.4)
	16–19	Harmful level	4 (4.0)	1 (1.0)	4 (4.0)	10 (10.0)	6 (6.0)	25 (5.0)
	≥20	Dependence likely	9 (9.0)	6 (6.0)	4 (4.0)	9 (9.0)	5 (5.0)	33 (6.6)
Smartphone addiction	15–39	Normal user	85 (85.0)	91 (91.0)	69 (69.0)	94 (94.0)	98 (98.0)	437 (87.4)
	40–43	At-risk	5 (5.0)	4 (4.0)	0 (0.0)	0 (0.0)	1 (1.0)	10 (2.0)
	≥44	High Risk	5 (5.0)	3 (3.0)	0 (0.0)	2 (2.0)	1 (1.0)	11 (2.2)
		Missing	5 (5.0)	2 (2.0)	31 (31.0)	4 (4.0)	0 (0.0)	42 (8.4)
Problem gambling	0	Non-problem	88 (88.0)	95 (95.0)	94 (94.0)	90 (90.0)	92 (92.0)	459 (91.8)
	1–2	Low level	7 (7.0)	3 (3.0)	3 (3.0)	4 (4.0)	5 (5.0)	22 (4.4)
	3–7	Moderate level	2 (2.0)	1 (1.0)	3 (3.0)	3 (3.0)	2 (2.0)	11 (2.2)
	≥8	Problem gambling	3 (3.0)	1 (1.0)	0 (0.0)	3 (3.0)	1 (1.0)	8 (1.6)

Notes: The five groups comprised unemployed people (including university students who do not engage in economic activities, Group1), full-time homemakers (Group2), and workers in the primary (Group3), secondary (Group4), and tertiary industries (Group5).

**Table 3:** Comparison of variables between groups.

Variable	Mean	Group1 ^a^	Group2 ^b^	Group3 ^c^	Group4 ^d^	Group5 ^e^	F	*p*	Partial η^2^	Bonferroni
Alcohol use disorder	Observed M (SD)	5.99 (7.71)	4.45 (6.33)	4.60 (6.21)	8.50 (7.28)	6.12 (6.45)	5.70	<0.001 ***		
	Adjusted M (SE)	5.54 (0.69)	4.49 (0.68)	5.60 (0.76)	8.07 (0.69)	5.97 (0.68)	3.63	0.006 **	0.03	b < d
Smartphone addiction	Observed M (SD)	29.29 (7.82)	28.01 (7.19)	24.19 (4.33)	26.71 (6.77)	25.76 (6.30)	7.28	<0.001 ***		
	Adjusted M (SE)	28.47 (0.68)	28.29 (0.66)	25.92 (0.85)	26.04 (0.67)	25.71 (0.65)	4.08	0.003 **	0.04	a > e
Problem gambling	Observed M (SD)	0.55 (2.28)	0.19 (0.99)	0.16 (0.76)	0.55 (2.46)	0.24 (1.18)	1.35	0.251		
	Adjusted M (SE)	0.60 (0.17)	0.19 (0.17)	0.05 (0.19)	0.60 (0.17)	0.26 (0.17)	1.74	0.139	0.01	
Aggression	Observed M (SD)	52.30 (13.69)	46.26 (13.01)	48.74 (10.60)	49.36 (12.17)	48.08 (9.38)	1.83	0.121		
	Adjusted M (SE)	52.04 (1.23)	49.28 (1.19)	49.27 (1.33)	49.15 (1.22)	48.00 (1.19)	1.53	0.193	0.01	
QOL	Observed M (SD)	3.29 (0.59)	3.34 (0.51)	3.35 (0.52)	3.41 (0.52)	3.44 (0.42)	1.26	0.283		
	Adjusted M (SE)	3.24 (0.05)	3.35 (0.05)	3.48 (0.06)	3.35 (0.05)	3.43 (0.05)	2.71	0.029 *	0.02	
Physical health	Observed M (SD)	13.80 (3.04)	14.34 (2.36)	13.84 (2.56)	14.33 (2.14)	14.32 (2.52)	1.21	0.306		
	Adjusted M (SE)	13.53 (0.26)	14.36 (0.25)	14.45 (0.28)	14.07 (0.25)	14.23 (0.25)	1.85	0.118	0.02	
Psychological health	Observed M (SD)	12.46 (2.84)	13.02 (2.51)	13.48 (2.46)	13.48 (2.61)	13.63 (2.28)	3.46	0.008 **		
	Adjusted M (SE)	12.24 (0.26)	13.04 (0.25)	13.95 (0.28)	13.27 (0.26)	13.56 (0.26)	5.51	<0.001 ***	0.04	a < c, d, e
Social relationships	Observed M (SD)	13.24 (3.51)	14.08 (1.87)	13.57 (2.75)	14.05 (2.74)	13.29 (3.49)	1.88	0.112		
	Adjusted M (SE)	12.94 (0.30)	14.11 (0.29)	14.24 (0.32)	13.76 (0.29)	13.19 (0.29)	3.39	0.009 **	0.03	a < b, a < c
Environment	Observed M (SD)	12.41 (3.02)	12.47 (2.28)	12.81 (2.13)	12.67 (2.75)	12.86 (2.60)	0.59	0.668		
	Adjusted M (SE)	12.15 (0.26)	12.49 (0.25)	13.37 (0.28)	12.42 (0.26)	12.77 (0.25)	2.59	0.036 *	0.02	a < c

Notes: M = mean; SD = standard deviation; SE = standard error; * *p* < 0.05; ** *p* < 0.01; *** *p* < 0.001; ^a^ unemployed including university students who do not engage in economic activities; ^b^ full-time homemakers; ^c^ workers in the primary industry; ^d^ workers in the secondary industry; ^e^ workers in the tertiary industry

**Table 4:** Correlations of variables by group.

Group	Variable	Pearson Correlation Coefficient (*p*)				
		1	2	3	4	5
Group1	1. Alcohol use disorder	1				
	2. Smartphone addiction	−0.02 (0.843)	1			
	3. Problem gambling	0.16 (0.120)	0.07 (0.483)	1		
	4. Aggression	0.18 (0.074)	0.12 (0.248)	0.24 (0.017)	1	
	5. QOL	−0.36 (<0.001) ***	0.06 (0.578)	−0.13 (0.224)	−0.27 (0.008) **	1
Group2	1. Alcohol use disorder	1				
	2. Smartphone addiction	0.30 (0.003) **	1			
	3. Problem gambling	0.30 (0.002) **	0.31 (0.002) **	1		
	4. Aggression	0.24 (0.019)	0.39 (<0.001) ***	0.01 (0.938)	1	
	5. QOL	0.15 (0.144)	−0.08 (0.434)	0.08 (0.455)	−0.38 (<0.001) ***	1
Group3	1. Alcohol use disorder	1				
	2. Smartphone addiction	0.13 (0.288)	1			
	3. Problem gambling	0.43 (<0.001) ***	0.12 (0.308)	1		
	4. Aggression	0.31 (0.002) **	0.19 (0.111)	0.17 (0.098)	1	
	5. QOL	0.14 (0.154)	−0.26 (0.028)	−0.00 (0.977)	−0.13 (0.204)	1
Group4	1. Alcohol use disorder	1				
	2. Smartphone addiction	0.27 (0.009) **	1			
	3. Problem gambling	0.03 (0.804)	0.09 (0.407)	1		
	4. Aggression	0.34 (0.001) **	0.31 (0.002) **	0.19 (0.067)	1	
	5. QOL	−0.23 (0.025)	−0.29 (0.004) **	−0.28 (0.005) **	−0.32 (0.002) **	1
Group5	1. Alcohol use disorder	1				
	2. Smartphone addiction	0.12 (0.239)	1			
	3. Problem gambling	0.10 (0.312)	0.08 (0.407)	1		
	4. Aggression	0.32 (0.001) **	0.17 (0.100)	0.39 (<0.001) ***	1	
	5. QOL	−0.07 (0.530)	−0.21 (0.039)	−0.10 (0.328)	−0.23 (0.027)	1

Notes: ** *p* < 0.01; *** *p* < 0.001.

## Data Availability

Restrictions apply to the availability of these data. Data were obtained from Gunsan AMC and are available with the permission of Gunsan AMC.
